# 501. Implementation and Outcomes of a Program to Coordinate and Administer Monoclonal Antibody Therapy to Long-Term Care Facility Residents with COVID-19

**DOI:** 10.1093/ofid/ofab466.700

**Published:** 2021-12-04

**Authors:** Andrew B Watkins, Lisa M Brand, Michelle Schwedhelm, Heather L Jensen, Brandon Scott, Dan K German, Kyle P Strand, Ishrat Kamal-Ahmed, James Lawler, M Salman Ashraf

**Affiliations:** 1 Nebraska Medicine, Omaha, Nebraska; 2 UNMC, Yutan, Nebraska; 3 Region VII Disaster Health Response Ecosystem (R7DHRE), Nebraska Medicine/University of Nebraska Medical Center, Omaha, Nebraska; 4 Great Plains Health, North Platte, Nebraska; 5 Community Pharmacy Services, Gretna, Nebraska; 6 Nebraska Department of Health and Human Services, Lincoln, Nebraska; 7 Division of Public Health, Nebraska Department of Health and Human Services, Lincoln, Nebraska; 8 University of Nebraska Medical Center, Omaha, Nebraska

## Abstract

**Background:**

Long-term care facility (LTCF) residents are at increased risk of severe COVID-19, with CMS data indicating > 20% mortality. BLAZE-1 trial noted lower hospitalization rates in high-risk patients receiving monoclonal antibody (mAb) vs placebo (4.2% vs 14.6%) for mild to moderate infections, making it a treatment option for LTCF residents; however, many LTCF lack staff to prepare and administer mAb therapy. To address this need, Region VII Disaster Health Response Ecosystem (R7DHRE) coordinated via NE Medical Emergency Operations Center (NEMEOC) an ASPR pilot project to facilitate infusion of COVID-19 mAb therapeutics for LTCF residents in the state.

**Methods:**

R7DHRE partnered with Great Plains Health, Nebraska DHHS, Nebraska Antimicrobial Stewardship Assessment and Promotion Program (ASAP) and Infection Control Assessment and Promotion Program (ICAP) to surveil cases in the state, establish distribution/administration pathways, and educate providers on mAb therapeutics. A multi-hub-and-spoke model was created to allow LTCF to work with regional hospitals or pharmacy services to administer drug in their facilities, reducing time to therapy and transmission risk associated with patient transport. A centralized request process was created using a REDCap platform and verification of patient eligibility by ASAP. This request link, informational documents, fact sheets, and custom-built order form templates were hosted on a dedicated ASAP webpage, and details were shared during weekly ICAP LTCF webinars. Outcomes data, including 14- and 28-day COVID-related hospitalizations and mortality, were collected using databases from Nebraska Health Information Initiative and Nebraska DHHS.

**Results:**

Through this program, 513 doses were administered to LTCF residents. Average time from symptom onset to infusion was 2.6 days. COVID- related hospitalization and mortality rates were lower than previously reported for LTCF residents (Table 1).

Table 1. Debographics and Outcomes of mAb Infusions

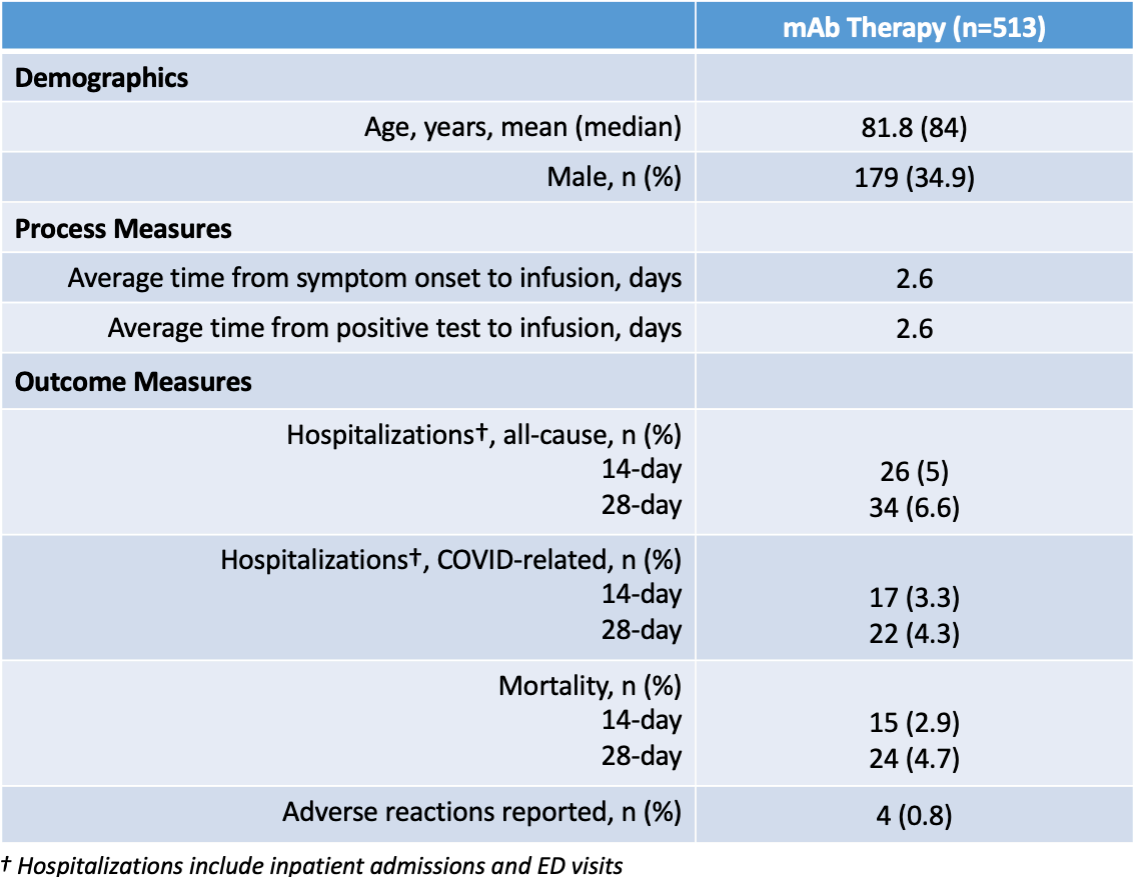

**Conclusion:**

By utilizing existing relationships with LTCFs in the region, we established a program to promptly distribute, prepare, and administer monoclonal antibody therapy to LTCF residents in need, preventing COVID-related hospitalizations and deaths.

**Disclosures:**

**James Lawler, MD, MPH, FIDSA**, **Kinsa Health** (Advisor or Review Panel member)**Takeda Pharmaceuticals** (Advisor or Review Panel member) **M. Salman Ashraf, MBBS**, **Merck & Co. Inc** (Grant/Research Support, I have recieved grant funding for an investigator initiated research project from Merck & Con. Inc. However, I do not see any direct conflict of interest related to the submitted abstract)

